# Screening and validation of reference genes in *Dracaena cochinchinensis* using quantitative real-time PCR

**DOI:** 10.1038/s41598-024-52754-5

**Published:** 2024-03-14

**Authors:** Shixi Gao, Junxiang Peng, Mei Rong, Yang Liu, Yanhong Xu, Jianhe Wei

**Affiliations:** 1 Key Laboratory of Bioactive Substances and Rescoures Utilization of Chinese Herbal Medicine, Ministry of Education & National Engineering Laboratory for Breeding of Endangered Medicinal Materials, Institute of Medicinal Plant Development, Chinese Academy of Medical Sciences and Peking Union Medical College, Beijing, China; 2 Hainan Procincial Key Laboratory of Resources Conservation and Development of Southern Medicine & Key Laboratory of State Administration of Traditional Chinese Medicine for Agarwood Sustainable Utilization, Hainan Branch of the Institute of Medicinal Plant Development, Chinese Academy of Medical Sciences and Peking Union Medical College, Hainan, China

**Keywords:** Molecular biology, Plant sciences

## Abstract

Dragon's blood, the red resin derived from the wounded *Dracaena*, is a precious traditional medicine used by different culture. *Dracaena cochinchinensis* is one of the main species of *Dracaena*, and is the endangered medicinal plants in China. The vulnerable status severely limits the medicinal value and wide application of dragon’s blood. Therefore, it’s essential to analyze the mechanisms that form dragon’s blood in order to increase artificial production. To clarify the mechanisms forming dragon’s blood, understanding gene expression in the flavonoid biosynthesis pathway is the foundation. However, reference genes of *D. cochinchinensis* haven’t been analyzed. In this study, expression profiles of seven commonly used housekeeping genes (*Actin*, *α-EF*, *UBC*, *β-tubulin*, *18S*, *GAPDH*, *His*) were evaluated by using quantitative real-time PCR combined with the algorithms geNorm, NormFinder, BestKeeper, and RefFinder. On the basis of overall stability ranking, the best reference genes were the combinations *β-tubulin* +*UBC* for wounded stems and *α-EF* +*18S* + *Actin* for different organs. Reliability of the recommended reference genes was validated by normalizing relative expression of two key enzyme genes *PAL1* and *CHI1* in the flavonoid biosynthesis pathway. The results provide a foundation to study gene expression in future research on *D. cochinchinensis* or other *Dracaena.*

## Introduction

*Dracaena cochinchinensis* (Lour.) S.C. Chen is an evergreen arbor in the family Asparagaceae. After injury, the stems secrete a type of red resin called dragon’s blood. Dragon’s blood has many pharmacological properties, including anti-inflammatory, antitumor, antibacterial, and hypoglycemic and hypolipidemic activities^1–5^, and is suitable for anorectal, orthopedic, and dermatological diseases^6–9^. Historically, dragon’s blood has also been used worldwide in cosmetics and dyes^10^. As research progresses, dragon’s blood use is increasing, leading to expanding market demand. However, a long growth cycle, climate change, long-term resin harvest, and other factors have reduced populations of dragon blood trees worldwide. Four species of the dragon tree group (*D. cinnabari*, *D. draco*, *D. ombet*, and *D. serrulata*) are listed in the International Union for Conservation of Nature Red List^11^. *Dracaena cambodiana* and *D. cochinchinensis* populations, mainly distributed in Asian countries such as China, Vietnam, and Laos, are also sharply decreasing^12^. Moreover, *D. cochinchinensis*, the original plant of dragon’s blood stipulated in the National Drug Standard of China, is listed in the List of National Key Protected Wild Plants of China. The listing is the second highest grade for national protection and prohibits the harvest of such endangered species^13^. Because of increasing demand for dragon’s blood and the endangered status of these valuable species, adequate measures must be developed to maintain and use dragon’s blood resources^14^.

Production of dragon’s blood can be increased by artificial trauma^15^ and by microbial, exogenous hormone, and small-molecule chemical induction^16–18^. Cui et al^19^ found that two fungi, *Fusarium thapsinum (BJDC01)* and *Septoria arundinacea* (*BJDC05)*, promoted the accumulation of five major components in dragon’s blood, creating conditions to artificially induce production of dragon’s blood. In addition, Yang et al.^17^ found that application of GA(gibberellic acid), IAA(indole-3-acetic acid), BR(brassinosteroid), and KT(kinetin) increases yields of dragon’s blood. However, technology has not been developed for the industrial production of dragon’s blood, primarily because the mechanism of wound-induced production is not very clear. Phytochemical studies indicate that flavonoids are the main active components of dragon’s blood, whereas in fresh stems, steroid compounds are the most abundant secondary metabolites^20^. The clear differences in flavonoid and steroid contents between resin and fresh wood demonstrate that wounding induces flavonoid production in dragon’s blood. Flavonoids are associated with plant defense against pathogens and microbes and absorption of free radicals and ultraviolet light^21–25^. Thus, the formation of dragon’s blood is a mechanism by which a dragon tree defends itself^12,26,27^. The main active substances in dragon’s blood are dihydrochalcone analogues such as loureirin A and loureirin B^28^ Therefore, studying the expression of key enzyme genes in the flavonoid biosynthetic pathway can be used to analyze the mechanisms of dragon’s blood production. Sun et al.^29^ found that three days after trauma, the blood trees began to synthesize large amounts of flavonoids that significantly upregulate the expression of key enzyme genes in the flavonoid biosynthetic pathway, such as *C4H, CHS*, and *CHI*. Transcriptome data of *D. cambodiana* indicated that most genes involved in flavonoid biosynthesis and transport are upregulated in stems after injecting an inducer, consistent with the accumulation of flavonoids^30^. Recently, the chromosome-level genome assembly of *D. cochinchinensis* was achieved, and by obtaining its gene dictionary, molecular mechanisms underlying longevity and formation of dragon’s blood in *D. cochinchinensis* have been preliminarily revealed^31^. Simultaneously, in combined transcriptome and metabolome analyses, a series of key enzyme and regulatory genes related to wound-induced formation of dragon’s blood were also identified^31,32^. Those results are valuable resources and substantially broaden the scope for studies on *D. cochinchinensis.* Because dragon’s blood is formed by wounding, regulation of the expression of many related genes will be at the transcriptional level*.* Thus, analyzing gene expression is crucial in exploring molecular mechanisms of flavonoid biosynthesis and formation of dragon’s blood and is also the first step in increasing sustainable use of the natural resource.

Nowadays, quantitative real-time PCR (qPCR) is the tool most frequently used to determine the mRNA levels in different biological systems. The advantages of qPCR include cost-effectiveness, specificity, and sensitivity when compared with traditional semi-quantitative, Northern hybridization, and current transcriptome sequencing technologies. However, in qPCR experiments, sample size, RNA quality, and primer specificity can affect the stable expression of target genes^33^. Normalization is an important step in qPCR analysis, which requires correction of target gene data according to expression of internal reference genes. Therefore, reference genes that are stably expressed need to be selected to mitigate potential sources of error and to obtain accurate relative expression of target genes.

Reference genes should be stably expressed under different conditions and in different organs or organs and developmental stages. Housekeeping genes are usually selected as reference genes because of relatively consistent expression throughout plant life or in response to changes in the external environment. However, an increasing number of recent studies have shown that the frequently used reference genes are not always stable under different conditions. In *Cyclocarya paliurus*, *18S rRNA* was the most stable reference gene in different organs^34^. In *Cypripedium japonicum*, *PP2A3* (Protein phosphatase 2A-3) was the most suitable reference genes in different organs, but *TUBB3* (tubulin beta 3 class III) and *UBC2* (Ubiquitin conjugating enzyme 2) had the most stable expression at different stages of seed development^35^. It is also common to screen reference genes for crop plants^36–38^. Thus, the optimal internal reference gene is not universal. Consequently, different reference genes need to be selected for different conditions to ensure the accuracy of target gene expression. At present, related studies are widely combined with omics-data, and four softwares, geNorm, NormFinder, BestKeeper and RefFinder, are used to screen internal reference genes^39–42^.

To date, reference genes in *D. cochinchinensis* have not been screened. In this study, seven candidate reference genes were selected based on previous transcriptome data, and gene expression stability was assessed using qPCR combined with geNorm, NormFinder, BestKeeper, and RefFinder software. The applicability of selected reference genes was evaluated by examining the expression of *PAL1* and *CHI1*, two key enzyme genes in the flavonoid biosynthesis pathway. The work will help to further elucidate the molecular mechanisms underlying the formation of dragon’s blood.

## Results

### Detection of primer specificity

**Figure 1:**
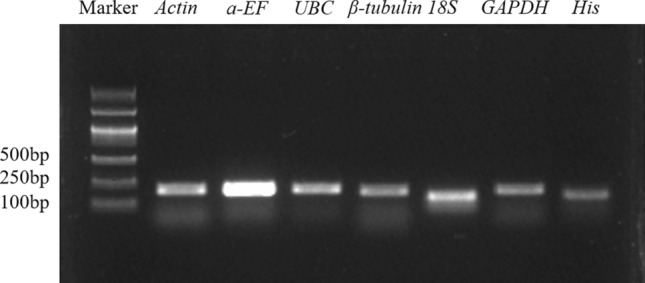
Agarose gel electrophoresis of candidate reference genes (original gel is presented in Supplementary Fig. S1 online).

Two to three pairs of primers were designed for each candidate reference gene, and the most appropriate was selected based on the PCR products and qPCR melting curves. Agarose gel electrophoresis showed that all selected primer pairs amplified a single PCR product with the expected size (Fig. 1**)**. In the qPCR experiments, the melting curve for each reference gene was a single peak, which also demonstrated the specificity of the primers.

### Expression profiles of candidate reference genes

**Figure 2:**
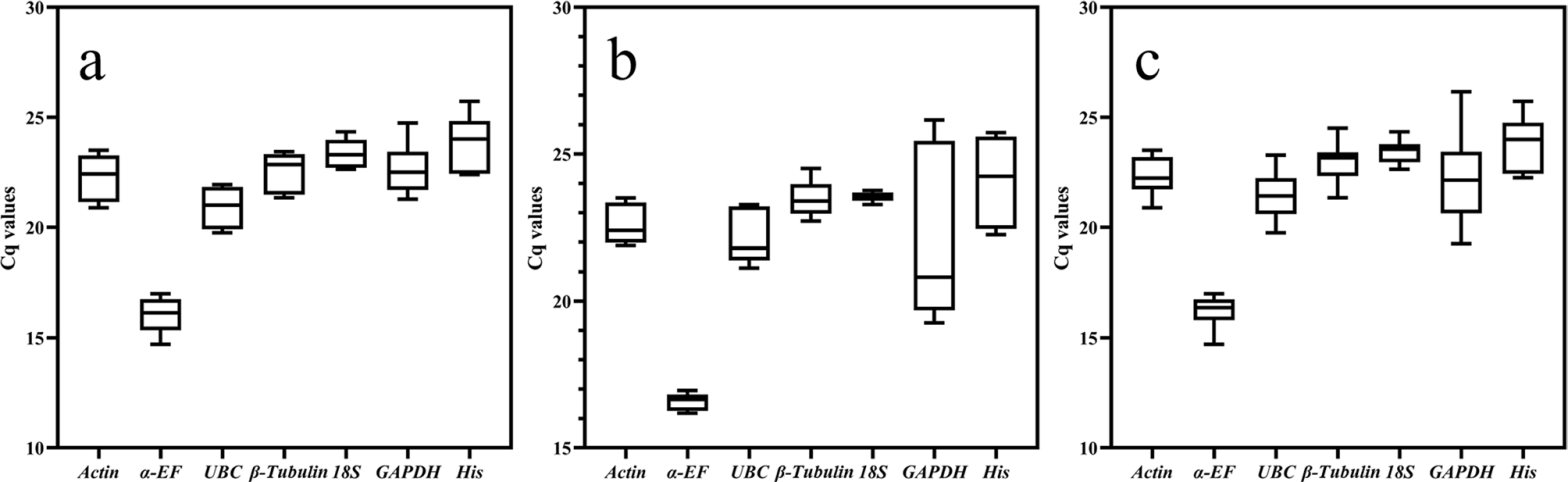
Cq values of candidate reference genes. Box plots of Cq values show median values as lines across the box. Lower and upper boxes indicate the 25th percentile to the 75th percentile. Whiskers represent maximum and minimum values. (**a**) Samples of wounded stems at different times (0, 6, and 24 h and 3, 10, and 30 d). (**b**) Samples of different organs (roots, leaves, flowers, fruits and stems). (**c**) All samples. Three replicates per sample.

**Figure 3:**
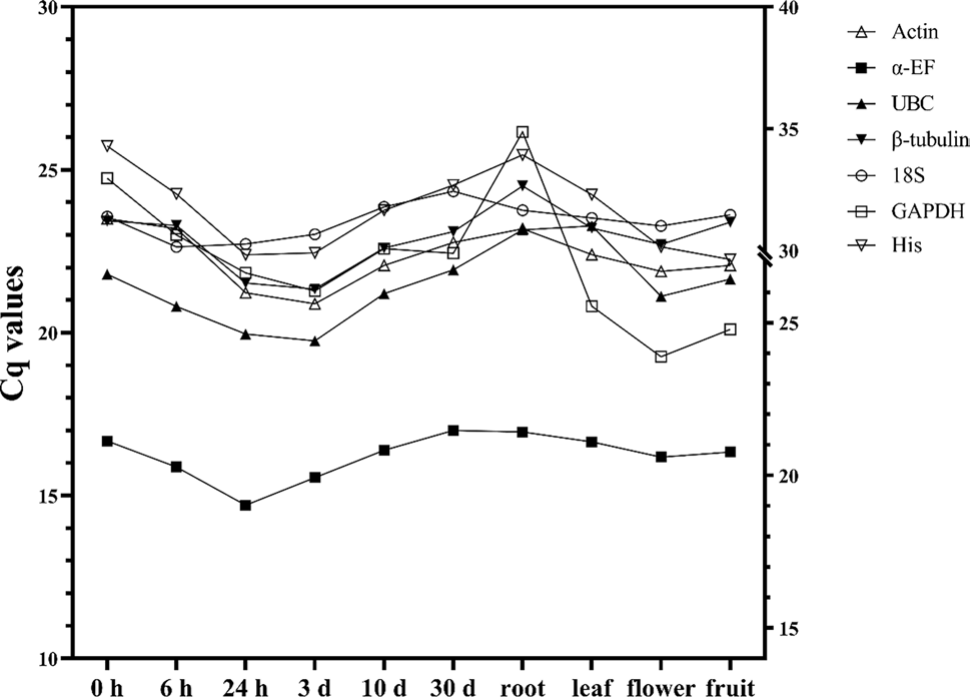
Average Cq value of candidate reference genes in each sample. Horizontal coordinate: Samples of wounded stems at different times and samples of different organs of *Dracaena cochinchinensis.*

The Cq value obtained from qPCR experiments was the number of cycles that the fluorescent signal underwent when it reached a specific threshold. The lower the Cq value was, the higher the gene expression in the sample. The Cq values analyzed using raw expression data from all samples ranged from 14.71 to 26.16 (Figs. 2 and 3). The highest transcript level was for *α-EF*, with Cq values ranging from 14.71 to 17.00. Expression levels of the other candidate reference genes were similar, with Cq values of 20.89–23.50 for *Actin*, 19.75–23.28 for *UBC*, 21.35–24.51 for *β-tubulin*, 22.64–24.34 for *18S*, 19.26–26.16 for *GADPH*, and 22.25–25.73 for *His*.

### Stability analysis of candidate reference genes

To minimize the bias generated by the assumptions underlying each evaluation method, the software programs geNorm, NormFinder, BestKeeper, and RefFinder were used to assess and rank the expression stability of the candidate reference genes.

#### GeNorm analysis

In geNorm analysis, the expression stability of each internal reference gene was measured by M with a recommended cutoff value of 1.5. The smaller the M value was, the better the stability of the internal reference gene. In total samples and those of wounded stems, *Actin* and *β-tubulin* were the most stable reference genes, with M values of 0.223 and 0.474, respectively (Table 1). In samples of different organs, *α-EF* and *18S* were the most stable reference genes, both with M values of 0.197. In total samples and those of different organs, *GAPDH* was the most unstable gene, with the highest M values. In samples of wounded stems, *18S* was the most unstable gene, with an M value 0.677.

**Table 1:** Candidate reference genes ranked by geNorm. *α-EF*: α-Elongation factor; *His*: Histone; *UBC*: Ubiquitin conjugating enzyme; *18S*: 18S rRNA; *GAPDH*: Glyceraldehyde 3-phosphate dehydrogenase.

Ranking	All samples		Samples of wounded stems at different times		Samples of different organs	
	Candidate reference genes	Stablity value	Candidate reference genes	Stablity value	Candidate reference genes	Stablity value
1	*Actin*	0.474	*Actin*	0.223	*α-EF*	0.197
2	*β-tubulin*	0.474	*β-tubulin*	0.223	*18S*	0.197
3	*α-EF*	0.553	*His*	0.389	*β-tubulin*	0.373
4	*18S*	0.620	*UBC*	0.469	*Actin*	0.465
5	*UBC*	0.689	*α-EF*	0.531	*UBC*	0.609
6	*His*	0.774	*GAPDH*	0.592	*His*	0.831
7	*GAPDH*	1.063	*18S*	0.677	*GAPDH*	1.311

**Figure 4:**
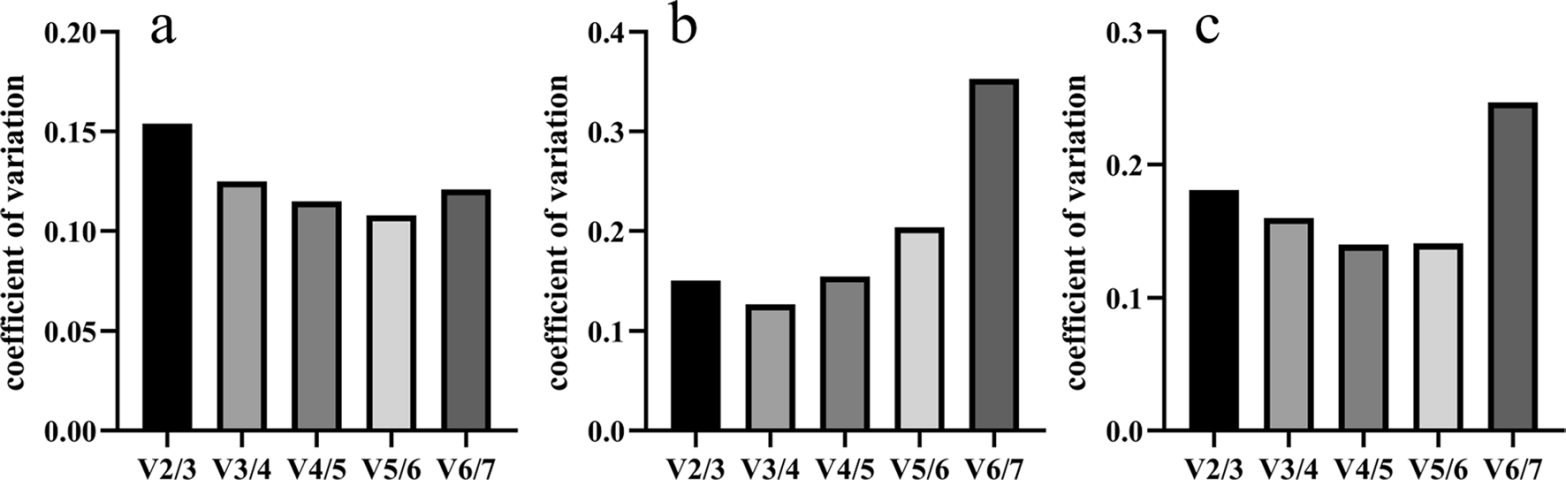
Pairwise variation analysis of candidate reference genes. (**a**) Samples of wounded stems at different times after wounding. (**b**) Samples of different organs. (**c**) All samples.

GeNorm also used pairwise difference analysis to obtain the optimal number of internal reference genes in different samples. In samples of wounded stems at different times, all values of Vn/n + 1 except V2/3 were less than 0.15, with V5/6 the smallest (Fig. 4A). According to the principle of standardized factor difference analysis, five combinations of reference genes needed to be selected to correct the results of gene expression. In all samples, V4/5 and V5/6 had the smallest values, which were less than 0.15 (Fig. 4C). However, in a qPCR experiment, using too many reference genes is time-consuming and can even increase the error^43^. In general, choosing two to three stable reference genes to correct the results provides sufficient accuracy. Thus, for all samples and those of wounded stems, a combination of two to three reference genes would be recommended for the most accurate results. Similarly, in samples of different organs, only the value of V3/4 was less than 0.15, and therefore, the most suitable combination of internal reference genes was three (Fig. 4B).

#### NormFinder analysis

The principle of NormFinder is similar to that of geNorm, and it also ranked internal reference genes according to the stability value M. However, NormFinder can only screen for the single most suitable reference gene. The NormFinder program compared expression differences of candidate internal reference genes and also calculated expression differences between sample groups. According to NormFinder analysis, in wounded stems, *UBC* was the most stable reference gene, with an M value of 0.209 (Table 2). In the samples of different organs, *Actin* was the most stable reference gene, with an M value of 0.243. In all samples, *Actin* was also the most stable reference gene, with an M value of 0.281. Consistent with the geNorm software analysis of total and different tissue samples, *GAPDH* was the most unstable gene, and the highest M values indicated that it was unstable in expression and therefore unsuitable for use as a reference gene.

**Table 2:** Candidate reference genes ranked by NormFinder. *α-EF*: α-Elongation factor; *His*: Histone; *UBC*: Ubiquitin conjugating enzyme; *18S*: 18S rRNA; *GAPDH*: Glyceraldehyde 3-phosphate dehydrogenase.

Ranking	All samples		Samples of wounded stems at different times		Samples of different organs	
	Candidate reference genes	Stablity value	Candidate reference genes	Stablity value	Candidate reference genes	Stablity value
1	*Actin*	0.281	*UBC*	0.209	*Actin*	0.243
2	*β-tubulin*	0.395	*β-tubulin*	0.229	*β-tubulin*	0.393
3	*α-EF*	0.492	*Actin*	0.402	*a-EF*	0.621
4	*His*	0.515	*a-EF*	0.455	*His*	0.750
5	*UBC*	0.755	*His*	0.469	*UBC*	0.821
6	*18S*	0.787	*GAPDH*	0.690	*18S*	0.858
7	*GAPDH*	1.715	*18S*	0.825	*GAPDH*	2.457

To summarize, *Actin* and *β-tubulin* were the most stable reference genes in total samples and those of different organs; whereas *UBC* and *β-tubulin* were the most stable reference genes in wounded stem samples. In different samples, *GAPDH* was the least stable, indicating it was not suitable as a reference gene.

#### BestKeeper analysis

BestKeeper validated the stability of candidate reference genes by calculating the standard deviations (SD). In all sample groups, *18S* was the most stable reference gene, with SD values of 0.56, 0.12, and 0.41, respectively (Table 3). However, in total samples and those of different organs, SDs of *His* and *GAPDH* were greater than 1, indicating those genes could not be used as reference genes. Collectively, in samples of wounded stems at different times, stability ranking of reference genes was the following: *18S* > *α-EF* > *UBC* > *β-tubulin* > *GAPDH* > *Actin* > *His*. In samples of different organs, gene stability was ranked as follows: *18S* > *α-EF* > *β-tubulin* > *Actin* > *UBC* > *His* > *GAPDH*. In total samples, stability ranking of genes was the following: *18S* > *α-EF* > *Actin* > *β-tubulin* > *UBC* > *His* > *GAPDH*.

**Table 3:** Candidate reference genes ranked by BestKeeper. *α-EF*: α-Elongation factor; *His*: Histone; *UBC*: Ubiquitin conjugating enzyme; *18S*: 18S rRNA; *GAPDH*: Glyceraldehyde 3-phosphate dehydrogenase.

Ranking	All samples				Samples of wounded stems at different times				Samples of different organs			
	Candidate reference genes	SD	CV^a^	r^b^	Candidate reference genes	SD	CV	r	Candidate reference genes	SD	CV	r
1	*18S*	0.41	1.76	0.581	*18S*	0.56	2.41	0.614	*18S*	0.12	0.50	0.758
2	*α-EF*	0.52	3.22	0.824	*α-EF*	0.65	4.08	0.888	*α-EF*	0.24	1.45	0.969
3	*Actin*	0.69	3.10	0.897	*UBC*	0.74	3.52	0.962	*β-tubulin*	0.42	1.80	0.849
4	*β-tubulin*	0.69	3.03	0.876	*β-tubulin*	0.74	3.30	0.950	*Actin*	0.59	2.61	0.928
5	*UBC*	0.90	4.18	0.801	*GAPDH*	0.82	3.60	0.853	*UBC*	0.81	3.66	0.651
6	*His*	1.07	4.51	0.940	*Actin*	0.88	3.95	0.929	*His*	1.29	5.38	0.938
7	*GAPDH*	1.56	7.03	0.776	*His*	0.99	4.14	0.983	*GAPDH*	2.59	11.65	0.975

^a^coefficient of variation, indicates the degree of data dispersion. The larger the CV value is, the more discrete the data. ^b^correlation coefficient, indicates the correlation between variables. The larger the absolute value of *r* is, the greater the correlation.

#### RefFinder analysis

Based on the ranking of each program above, RefFinder assigned appropriate weights to individual genes and calculated the geometric mean of the weights to obtain the overall ranking. In the comprehensive analysis (Table 4), in wounded stem samples, stability of candidate reference genes was ranked as follows: *β-tubulin* > *UBC* > *Actin* > *α-EF* > *18S* > *His* > *GAPDH*. In different tissue samples, stability of candidate reference genes was ranked as follows: *α-EF* > *18S* > *Actin* > *β-tubulin* > *UBC* > *His* > *GAPDH*. The combined ranking was *Actin* > *β-tubulin* > *α-EF* > *18S* > *His* > *UBC* > *GAPDH*.

**Table 4:** Candidate reference genes ranked by RefFinder. *α-EF*: α-Elongation factor; *His*: Histone; *UBC*: Ubiquitin conjugating enzyme; *18S*: 18S rRNA; *GAPDH*: Glyceraldehyde 3-phosphate dehydrogenase.

Ranking	All samples		Samples of wounded stems at different times		Samples of different organs	
	Candidate reference genes	Geometric value	Candidate reference genes	Geometric value	Candidate reference genes	Geometric value
1	*Actin*	1.32	*β-tubulin*	1.68	*α-EF*	1.57
2	*β-tubulin*	2.21	*UBC*	2.21	*18S*	2.21
3	*α-EF*	2.45	*Actin*	2.71	*Actin*	2.38
4	*18S*	3.31	*α-EF*	3.56	*β-tubulin*	2.71
5	*His*	4.90	*18S*	4.30	*UBC*	5.00
6	*UBC*	5.23	*His*	4.79	*His*	5.42
7	*GAPDH*	7.00	*GAPDH*	5.73	*GAPDH*	7.00

To summarize, compared with other candidate reference genes, *Actin* and *β-tubulin* showed the highest stability in each sample of *D. cochinchinensis* and therefore were most suitable as reference genes for quantitative analysis of target gene expression. When analyzing samples of wounded stems at different times after wounding, highly stable *β-tubulin* and *UBC* should be selected as reference genes. When analyzing samples of different organs, *α-EF* and *18S* should be selected as reference genes.

### Validation of stability of reference genes

**Figure 5:**
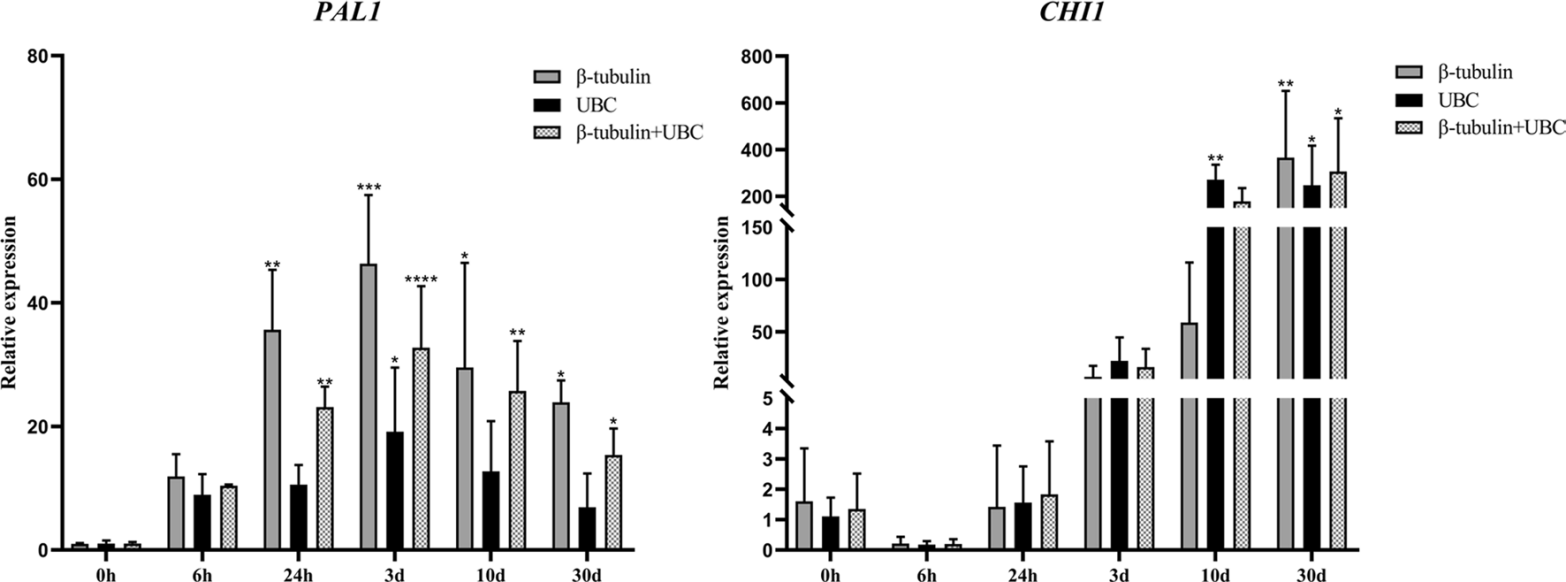
Expression levels of *PAL1* and *CHI1* at different times after wounding in stems of *Dracaena cochinchinensis*. Expression is shown of *PAL1* and *CHI1* with *UBC*, *β-tubulin,* and *UBC*+*β-tubulin* as reference genes. Bars indicate the standard deviation (SD) between biological replicates of each group. Three replicates per sample.*P ≤ 0.05; **P ≤ 0.01; ***P ≤ 0.001. *PAL1*: Phenylalanine ammonia-lyase 1; *CHI1*: Chalcone isomerase 1; *UBC*: Ubiquitin conjugating enzyme.

**Figure 6:**
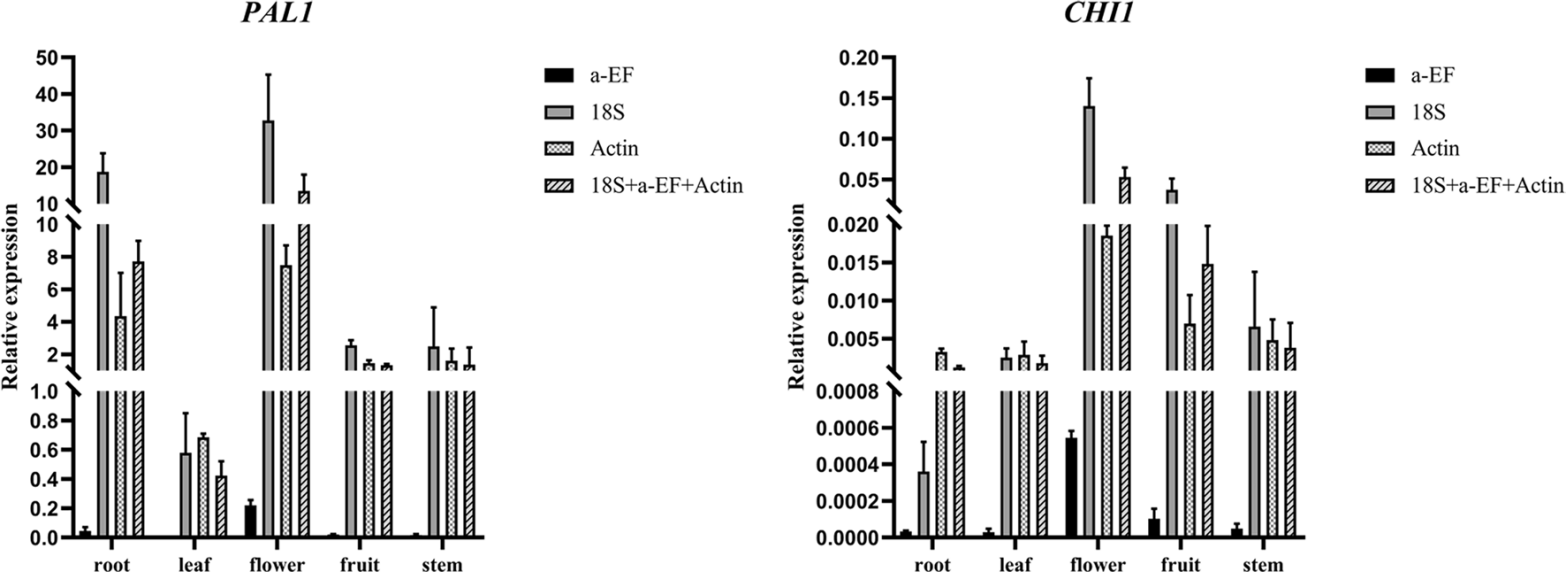
Expression levels of *PAL1* and *CHI1* in different organs of Dracaenacochinchinensis. Expression is shown of *PAL1* and *CHI1* with *α-EF*, *18S*, *Actin*, and *α-EF*+*18S*+*Actin* as reference genes. Bars indicate the standard deviation (SD) between biological replicates of each group. Three replicates per sample. *PAL1*: Phenylalanine ammonia-lyase 1; *CHI1*: Chalcone isomerase 1; *α-EF*: α-Elongation factor; *18S*: 18S rRNA.

To determine the accuracy and validate the confirmed reference genes, relative expression of the genes *CHI1* and *PAL1* was measured. The specificity of primers was detected by agarose gel electrophoresis(see Supplementary Fig. S3 online). To detect expression in wounded stems at different times, *β-tubulin*, *UBC*, and *β-tubulin* + *UBC* were used as internal reference genes, and to detect expression in different organs, *α-EF*, *18S*, *Actin*, and *α-EF*+*18S*+*Actin* were used as internal reference genes. In wounded stems, expression of *PAL1* gradually increased during the formation of dragon’s blood, reaching a maximum at 3 d post-wounding, and then decreased. Expression of *CHI1* increased from day three to day 30 but also initially decreased and reached a minimum value at 6 h post-wounding (Fig. 5). However, in both cases, the expression patterns were identical using *β-tubulin* or *UBC* or *β-tubulin*+*UBC* as the internal reference. Among the different organs, *PAL1* expression was highest in flowers and roots of *D. cochinchinensis*, whereas *CHI1* expression was highest in flowers and fruits (Fig. 6). With *α-EF*, *18S*, *Actin*, or *α-EF*+*18S*+*Actin* used as reference genes, expression patterns of the two target genes were generally consistent. Collectively, the results indicated that the reference genes screened in this study were stable and could be used to analyze gene expression in *D. cochinchinensis*.

Although expression trends were consistent, there were still some differences in transcript abundances of the target genes. The differences might be related to the level of expression of reference genes themselves. The most appropriate internal reference should be selected according to the specific conditions in the operation. In addition, combinations of internal reference genes are recommended to obtain the most accurate results.

## Discussion

Dragon’s blood is rich in flavonoids, and expression of key enzyme genes in the flavonoid biosynthesis pathway directly affects its yield and quality. Studying expression levels of target genes in wounded organs of *D. cochinchinensis* can increase understanding of the mechanism of resin formation. Therefore, selection of suitable reference genes is a prerequisite to obtain accurate and reliable quantitative data on target gene expression. Genes involved in forming cytoskeleton structure, such as *Actin*, *β-tubulin*, and *18S*, as well as those involved in biological metabolic processes, such as *GAPDH*, are often used as reference genes. However, those genes are not necessarily reliable in all situations.

In this study, seven commonly used housekeeping genes were tested as reference genes, including *Actin*, *α-EF*, *UBC*, *β-tubulin*, *18S*, *GAPDH*, and *His.* The stability of the seven internal reference genes were evaluated using four algorithms. Because of differences in algorithms and evaluation metrics, the results of analyses varied between software. For example, in geNorm, *Actin* and *β-tubulin* were the most stable reference genes in wounded stems, whereas in NormFinder and BestKeeper, *UBC* and *18S* were the most stable genes, respectively. However, whether in different organs or at different times following wounding, all programs indicated discarding *GAPDH* and *His* for use in normalization. Because the ranking of candidate reference genes was different in different programs, RefFinder was used to calculate the geometric means of the results of the three software to determine the overall stability of the seven candidate reference genes.

The geNorm software used the value of M as the criterion to determine the stability of a reference gene, but in contrast to NormFinder and BestKeeper, it also provided the optimum number of reference genes to suit specific experimental conditions. In this study, in samples of wounded stems at different times, all values of Vn/n + 1 except V2/3 were less than 0.15, with V5/6 the smallest, and therefore, according to the principle, a combination of five reference genes should be selected to correct gene expression results. However, in practice, using too many reference genes is time-consuming and can even increase the error^43^. Therefore, the general recommendation is to use two to three reference genes for pairing and combination.

According to RefFinder analysis, *β-tubulin* was the most stable reference gene in wounded stems, and *α-EF* was the most stable across different organs. In previous studies, *β-tubulin* was the most stable reference gene in different cultivars of *Brassica oleracea* L. var. *botrytis* L.^44^ but was less stable in different organs of *Sedum sarmentosum*^45^. By contrast, whereas *GAPDH* was the least stable reference gene in this study, it was the most stable in leaves of *Uncaria rhynchophylla* exposed to different shading times^46^. Such results are further indication that the expression stability of reference genes is influenced by differences in species, organs, and treatments.

Stability of the screened reference genes was validated using *PAL1* and *CHI1*, the two key enzymes in the flavonoid synthesis pathway. Expression trends of both genes were consistent when the screened genes were used separately as references, indicating that the screened internal reference genes were reliable. *PAL1* was activated within 6 h after wounding and reached maximum expression levels at 3 d, whereas *CHI1* was activated at 3 d after wounding and its expression level continued to increase during the study. Similar to and therefore verifying the finding in this research, Liu et al*.*^32^ found that two *PAL* genes were activated within 24 h of wounding and that one *CHI* gene was activated three to five days after wounding, according to clustering of expression patterns of genes annotated in the flavonoid biosynthetic pathway. Although when a single gene was used as reference, the expression trends of target genes were consistent with those when a combination of genes was used as reference, there was some variation in transcript abundances of the target genes. In particular, expression levels of genes with different internal references differed by two orders of magnitude in different tissue samples. The differences might be related to the expression levels of the reference genes themselves. Therefore, different statistical calculations should be used to determine the most suitable reference gene for different experimental conditions. On the basis of stability results, combinations of reference genes are recommended for use in qPCR.

**Figure 7:**
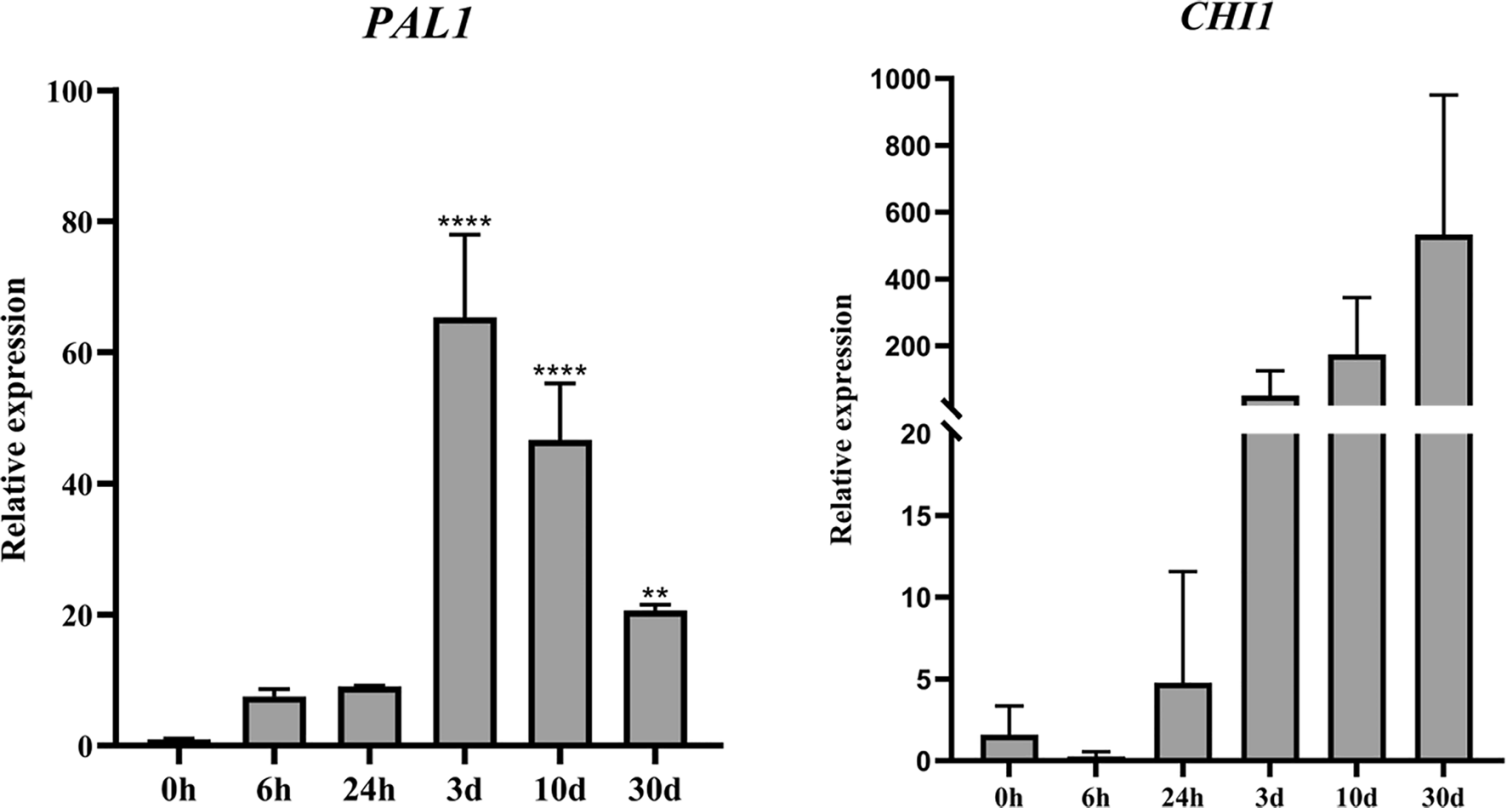
Expression levels of *PAL1* and *CHI1* in wounded stems of *Dracaena cochinchinensis*. Expression is shown of *PAL1* and *CHI1* in wounded stems with *18S* as the reference gene. Bars indicate the standard deviation (SD) between biological replicates of each group. Three replicates per sample. **P ≤ 0.01; ****P ≤ 0.0001. *PAL1*: Phenylalanine ammonia-lyase 1; *CHI1*: Chalcone isomerase 1; *18S*: 18S rRNA.

**Figure 8:**
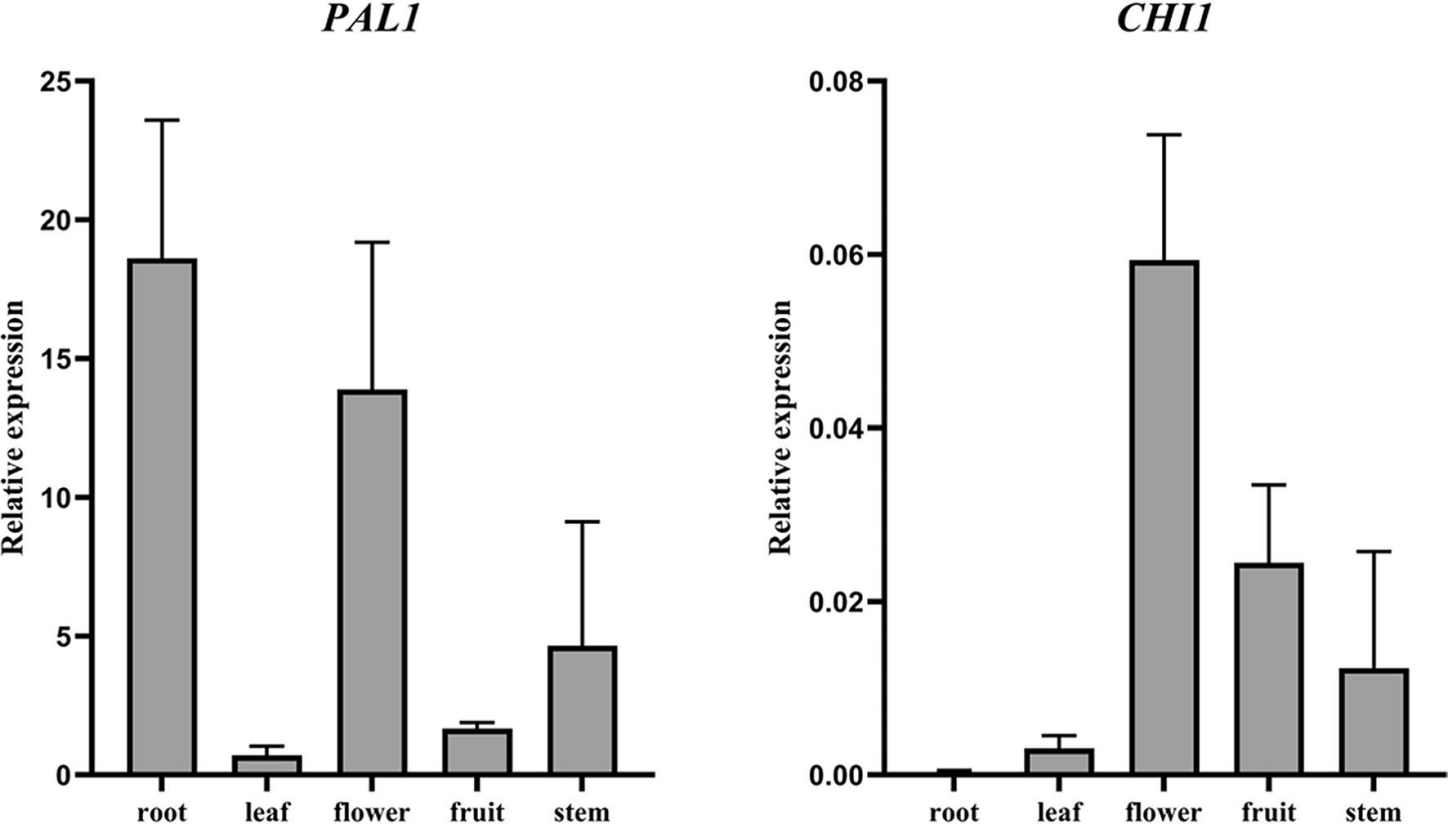
Expression levels of *PAL1* and *CHI1* in different organs of *Dracaena cochinchinensis*. Expression is shown of *PAL1* and *CHI1* in different organs with *β-tubulin* as the reference gene. Bars indicate the standard deviation (SD) between biological replicates of each group. Three replicates per sample. *PAL1*: Phenylalanine ammonia-lyase 1; *CHI1*: Chalcone isomerase 1.

Because the screened reference genes also ranked highly among all samples, the suitability of *18S* in wounded stems and *β-tubulin* in different organs was evaluated. Expression trends of *PAL1* and *CHI1* were consistent with those in the previous evaluation (Figs. 7 and 8), which indicated that the screened reference genes were suitable for different materials of *D. cochinchinensis.*

## Conclusions

Preliminary screening and validation of the reference genes of *D. cochinchinensis* were conducted. The best combination of reference genes in wounded stems at different times was *β-tubulin*+*UBC*; whereas the best combination in different tissue samples was *α-EF*+*18S*+*Actin*. In addition, *18S* can also be used as reference gene for wounded stems, and *β-tubulin* can also be used for different organs. This study provides a foundation for further analysis of gene expression and subsequent studies on the molecular mechanisms forming dragon’s blood.

**Table 5:** Primer details of candidate reference genes and target genes. The standard curve is detailed in Supplementary Fig. S2 online.

Gene symbol	Candidte reference gene	Primer sequence(5′ → 3′)	Amplicon length/bp	E(%)	R^2^	Slope	y intercept
*Actin-*Chr5.246	Actin	F:CCCTGAAAACTGCTCTGCTCTC	210	108.1	0.984	− 3.141	22.859
		R:CCTCAGGAAATCCCATCTCAAC					
*α-EF-*Chr6.259	α-Elongation factor	F:CTACAGGAGGCTCTTCCAGGTG	214	111.8	0.969	− 3.069	16.523
		R:CAGCAATGTGAGAGGTGTGGC					
*UBC-*Chr2.1570	Ubiquitin conjugating enzyme	F:TTCGCTGGGGGTGTGTTTC	213	100.4	0.990	− 3.312	24.037
		R:CGGGTTTGGGTCCGTAAGAAG					
*β-tubulin-*Chr4.2695	β-tubulin	F:TGAGCACCCTCCTGGTCGTA	195	119.7	0.990	− 2.926	22.418
		R:TGACCTCTGGACACCAACGG					
*18S-*Chr3.1083	18S rRNA	F:GCAGATGGAGGAGGATAGGGTAG	152	108.7	0.921	− 3.129	23.255
		R:TAAAAGCAGACTTCTCTCCCCC					
*GAPDH-*Chr4.1490	Glyceraldehyde 3-phosphate dehydrogenase	F:CCAACAAATCATCAAGGACATCAG R:ATGACACAAGCGATTCCATAGAGG	201	125.5	0.915	− 2.831	23.202
*His-*Chr6.1242	Histone	F:GCCAAGGCAACAGAAAACTCA	162	125.0	0.992	− 2.840	41.025
		R:ATCCATCACCCTCGTCACCTTA					
*CHI1-*Chr11.863	Chalcone isomerase 1	F:CACCACCACAAAGGCTTCACC	159	96.8	0.966	−3.400	45.944
		R: GCGAGTTCTCTGGCATTCTTTC					
*PAL1-*Chr1.1341	Phenylalanine ammonia-lyase 1	F: CTACATCGACGACCCTTGCAG	242	100.6	0.984	− 3.309	36.984
		R: ATGACCTGCATTCCTTGATCCTG					

## Methods

### Plant materials and treatments

*D. cochinchinensis* was analyzed in this study and the formal identification of the plant materials was undertaken by Mr.J.H. Wei. We got the permission to collect the plant samples and all methods were performed in accordance with the relevant guidelines and regulations. The materials for qPCR are organs from ten-year-old adult *D. cochinchinensis* trees growing in the germplasm bank at the Yunnan Branch of the Institute of Medicinal Plant Development, Chinese Academy of Medical Sciences, Jing-hong City (22.0058, 100.7885), China.

Three healthy *D. cochinchinensis* trees of similar age were selected, and roots, leaves, flowers, and fruits were sampled, with three biological replicates of each. A 3 cm long, 2 cm wide, 1 cm deep incision was cut in the trunk to cause wound stress using an alcohol-sterilized knife. The interval between incisions was > 5 cm to avoid an interaction effect. Scrape 1 g samples from the resin-forming area of each incision at different time points (0, 6, and 24 h and 3, 10, and 30 d). Three biological repeats at each time. After collection, samples were immediately frozen in liquid nitrogen and stored at − 80 °C for three years until extraction of RNA.

### RNA extraction and cDNA synthesis

Total RNA was extracted from each sample using an EASYspin Plus Plant RNA Kit (Aidlab, Beijing, China) according to the manufacturer’s instructions. The lysate in the kit was able to inactivate RNase in plant cells. Quality and concentration of RNA were measured using a Nanodrop 2000 spectrophotometer (Thermo Scientific, Shanghai, China), and RNA integrity was confirmed by 1% agarose gel electrophoresis. Only RNA with the required concentration and quality was reverse transcribed. With 1000 ng of RNA per sample as a template, cDNA was synthesized using a PrimeScript™ RT reagent Kit with gDNA Eraser (Perfect Real Time) Reverse Transcription Kit (TaKaRa, Beijing, China) according to the manufacturer’s instructions. The reaction was divided into two steps, firstly a total volume of 10 μl containing 2 μl of 5×gDNA Eraser Buffer, 1 μl gDNA Eraser, 1000 ng of RNA and variable volume of RNase free dH_2_O, at 42 °C for 2 min, followed by the addition of 4 μl 5× PrimeScript Buffer 2 (for Real Time), 1 μl PrimeScript RT Enzyme Mix I, 1 μl RT Primer Mix and 4 μl RNase free dH_2_O. The reaction procedure was 37 °C for 15 min, 85 °C for 5 s. The products were used in further experiments.

### Primer design and specificity detection

Based on the transcriptome data of *D. cochinchinensis*, seven candidate internal reference genes (*Actin*, *α-EF*, *UBC*, *β-tubulin*, *18S*, *GAPDH*, and *His*) were selected. The data has been deposited in the Genome Sequence Archive^47^ in the BIG Data Center, Beijing Institute of Genomics (BIG), Chinese Academy of Sciences, BioProject ID: PRJCA007701. Primers for candidate reference genes were designed according to primer design principles using Primer Premier 5.0 software (PREMIER Biosoft, Palo Alto, CA, USA) and then synthesized by Beijing New Times Zhonghe Technology Company (China). The cDNA from each sample was mixed evenly and used as a template for PCR amplification^48^. The template was diluted four times at five-fold dilution, and the qPCR reaction was performed to make a standard curve with concentrations and Cq values (Table). Reactions were prepared in a total volume of 10 μl containing 1 μl of diluted cDNA (100 ng/μl), 5 μl of 2× PrimeStar (TaKaRa), 3 μl of ddH_2_O, and 0.5 μl each of forward and reverse primers (10 μM). The PCR program was 94 °C for 5 min, 34 cycles of 94 °C for 30 s, 60 °C for 30 s, and 72 °C for 30 s, and 72 °C for 10 min. To analyze the specificity of primers, products were subjected to 1% agarose gel electrophoresis and run at 120 V for 15 min at room temperature. Gel images were obtained using Gel Doc XR+ (Bio-Rad, USA).

### Quantitative real-time PCR

Quantitative Real-Time PCR was performed using a CFX 96 Touch Real-Time PCR Detection System (Bio Rad, Beijing, China). Reactions were prepared in a total volume of 10 μl containing 5 μl of TransStart® Top Green qPCR SuperMix (TransGen, Beijing, China), 0.5 μl of cDNA (100 ng/μl), 3.5 μl of RNase free dH_2_O, and 0.5 μl each of forward and reverse primers (10 μM). The PCR program was 95 °C for 10 min and 45 cycles of 95 °C for 5 s, 60 °C for 15 s, and 72 °C for 20 s. Melting curves were analyzed at 65 °C to 95 °C after 45 cycles. Each qPCR analysis was performed in triplicate. RNase free dH_2_O was substituted for the template and other conditions were kept constant as a negative control (see Supplementary Table S1 online). Melting curve, melting temperature, and Cq value were output via CFX Maestro software v.2.3, where the Cq value was used to calculate the expression of reference genes in different samples.

### Data analyses

Stability rankings of candidate reference genes were obtained separately by geNorm v.3.5, NormFinder v.0.953, and BestKeeper v.1 software, and then, RefFinder 2020 was used to obtain a combined ranking of reference genes. The geNorm program calculated stability values (M) on the basis of the expression of each reference gene in different samples. The smaller the M value was, the better the stability of a reference gene. Only when the M value of a candidate gene was less than 1.5 was it considered suitable as a reference gene for qPCR. In addition, geNorm software calculated the optimal number of combinations of reference genes. In paired difference analysis, when Vn/n + 1 < 0.15, *n* internal reference genes should be selected for analysis of target gene expression; otherwise, *n* + 1 should be selected^49^. NormFinder and geNorm algorithms are similar, but NormFinder only screened for one stable reference gene. BestKeeper screened for internal reference genes by calculating the standard deviation (SD) based on the Cq values of the reference genes in different samples. The smaller the SD was, the better the stability of a candidate gene. When the SD was greater than 1, the gene was considered less stable and not used as a reference gene. RefFinder is an online web tool that calculated the geometric mean of the software results to obtain a comprehensive ranking of candidate reference genes.

Data were analyzed as follows: the minimum Cq value for each gene was subtracted from the other Cq values to obtain the ΔCq value, and then the 2^-ΔCq^ value was calculated using the exponential function (power function) in Microsoft® Excel 2019. The values in the 2^-ΔCq^ format for each sample were entered into geNorm and NormFinder software to obtain the stability value M and the stability order, respectively. The Cq values for each sample were entered into BestKeeper to obtain standard deviation (SD) data. Last, RefFinder was used to combine the rankings of the above three software to obtain a combined ranking and select the most suitable reference genes.

### Evaluation of suitable reference genes

The optimal reference genes selected were used as internal reference genes, and the stability was verified by detecting the relative expression of the key enzyme genes *PAL1* (Chr1.1341)and *CHI1*(Chr11.863) in the flavonoid biosynthesis pathway. The PCR reaction system was the same as *in Quantitative Real-Time PCR*. The relative expression of target genes in wounded stems was calculated according to the 2^−ΔΔCq^ method^50^. Healthy stems (0 h after incision) were the control and wounded stems (6 and 24 h and 3, 10, and 30 d after incision) were the experimental group. The relative expression of target genes in different organs was calculated according to the 2^−ΔCq^ method. Three biological repeats at each group. Each biological replicate had three technical replicates. Graphpad Prism v8.0.2 was used to perform one-way ANOVA analysis of the data (Table 5).

### Supplementary Information

Supplementary Information.

## Data Availability

The datasets used and analysed during the current study are available from the corresponding author on reasonable request.

## Abbreviations

*α-EF*: α-Elongation factor
*UBC*: Ubiquitin conjugating enzyme
*18S*: 18S rRNA
*GAPDH*: Glyceraldehyde 3-phosphate dehydrogenase
*His*: Histone
*P**AL1*: Phenylalanine ammonia-lyase 1
*C**HI1*: Chalcone isomerase 1

## References

[1] Li N, Ma Z, Li M, Xing Y, Hou Y (2014). Natural potential therapeutic agents of neurodegene-rative diseases from the traditional herbal medicine Chinese dragon’s blood. J. Ethnopharmacol..

[2] Meng XX (2017). Study on the antibacterial effect of dragon's blood and spider incense in vitro. West China J. Pharm. Sci..

[3] Wang H (2017). Flavonoids from artificially induced dragon's blood of *Dracaena cambodiana*. Fitoterapia..

[4] Wen F, Zhao X, Zhao Y, Lu Z, Guo Q (2016). The anticancer effects of Resina Draconis extract on cholangiocarcinoma. Tumour. Biol..

[5] Zhang L (2019). Advance of the chemical components and pharmacological effects of *Draconis Sanguis* and *Resina Draconis*. Chin. J. Mod. Appl. Pharm..

[6] Wang DY (2021). Clinical study of dragon's blood powder in promoting incision healing after operation of perianal abscess. J.. Mod. Med. Health.

[7] Yan S (2020). Observation of therapeutic effect of new medicine surgery and external application of dragon's blood powder on cervical spondylopathy of vertebral artery. Guide China Med..

[8] Zhang Y (2021). Effect of resina draconis gel on TNF-α, IL-6 level in serum and MFGE8 expression in the skin from rabbit’s iear acne model. Chin. J. Dermatovenereol..

[9] Zheng YF, Wang RF, Guo H (2017). Clinical observation on the treatment of 33 cases ofulcerative colitis with dragon's blood combined with sulfasalazine tablets local retention enema. Asia-Pacific Tradit. Med..

[10] Al-Fatimi M (2018). Ethnobotanical survey of *Dracaena cinnabari* and investigation of the pharmacognostical properties, antifungal and antioxidant activity of its resin. Plants..

[11] The IUCN Red List of Threatened Species. http://www.iucnredlist.org. Accessed 2 February (2023).

[12] Petr M (2020). What we know and what we do not know about dragon trees ?. Forests..

[13] Fu, L.G. Red Book of Chinese Plants–Rare and Endangered Plants. Book 1. Science Press, Beijing, China, 392 (1991).

[14] Petr M (2018). Growth dynamics of endemic *Dracaena cinnabari* Balf. F. of socotra Island suggest essential elements for a conservation strategy. Biologia..

[15] Cai XT, Xu ZF (1979). A study on the resource of Chinese dragon's blood. Acta Bot. Yunnanica..

[16] Wang H, Liu J, Wu J, Mei WL, Dai HF (2011). Flavonoids from *Dracaena cambodiana*. Chem. Nat. Compd..

[17] Yang XH, Zheng S, Wang XH (2014). Preliminary study on chemical inducers for dragon's blood formed by *Dracaena cambodiana*. Amino Acids Biotic. Resour..

[18] Zhu JH (2016). Transcriptome-wide identification and expression analysis of glutathione S-transferase genes involved in flavonoids accumulation in *Dracaena cambodiana*. Plant Physiol. Biochem..

[19] Cui JL, Wang CL, Guo SX, Xiao PG, Wang ML (2013). Stimulation of dragon's blood accumulation in Dracaena cambodiana via fungal inoculation. Fitoterapia.

[20] Liu Y (2021). Dragon's blood from dracaena worldwide: Species, traditional uses, phytochemistry and pharmacology. Am. J. Chin. Med..

[21] Giovanni A, Giovanni S, Stefano B, Massimiliano T (2009). Mesophyll distribution of 'antioxidant' flavonoid glycosides in leaves under contrasting sunlight irradiance. Ann. Bot..

[22] Giovanni A (2011). The biosynthesis of flavonoids is enhanced similarly by UV radiation and root zone salinity in L. vulgare leaves. J. Plant Physiol..

[23] Agati G, Tattini M (2010). Multiple functional roles of flavonoids in photoprotection. New Phytol..

[24] Landi M, Tattini M, Gould KS (2015). Multiple functional roles of anthocyanins in plant-environment interactions. Environ. Exp. Bot..

[25] Neill SO, Gould KS (2003). Anthocyanins in leaves: light attenuators or antioxidants?. Funct. Plant. Biol..

[26] Jura-Morawiec J, Tulik M (2016). Dragon’s blood secretion and its ecological significance. Chemoecology.

[27] Wang XH (2007). Flavones formed from xylem stem of *Dracaena cochinchinensis* by co-culture of fungus strain and bacteria strain. Nat. Prod. Res. Dev..

[28] Chen S, Wu SC, Zeng Y, Liu XM (2013). Anti-inflammatory and analgesic effects of total flavone extracted from dragon's blood and its analgesic mechanism exploration. Lishizhen Med. and Materia Medica. Res..

[29] Sun HF, Song MF, Zhang Y, Zhang ZL (2021). Transcriptome profiling reveals candidate flavonoid related genes during formation of dragon's blood from *Dracaena cochinchinensis* under conditions of wounding stress. J. Ethnopharmacol..

[30] Zhu JH (2016). De novo transcriptome characterization of *Dracaena cambodiana* and analysis of genes involved in flavonoid accumulation during formation of dragon’s blood. Sci. Rep..

[31] Xu YH (2022). A chromosome-level genome assembly for *Dracaena cochinchinesis* reveals molecular basis of its longevity and formation of dragon’s blood. Plant Commun..

[32] Liu Y (2022). Transcriptomics and metabolomics analyses reveal defensive responses and flavonoid biosynthesis of *Dracaena cochinchinensis* (Lour.) S. C. Chen under wound stress in natural conditions. Molecules.

[33] Sinha P (2015). Evaluation and validation of housekeeping genes as reference for gene expression studies in pigeonpea (*Cajanus cajan*) under drought stress conditions. PloS one.

[34] Qu YQ, Chen XL, Chen BQ, Fu XX (2021). Selection of reference genes for quantitative real-time PCR in *Cyclocarya paliurus*. Mol. Plant. Breed..

[35] Duan GM, Li TY, Tian M, Wang CX, Zhang Y (2021). Reference gene selection of real-time fluorescence quantitative PCR in *Cypripedium japonicum*. Nucl. Agr. Sci..

[36] Fernandez P (2011). Comparison of predictive methods and biological validation for qPCR reference genes in sunflower leaf senescence transcript analysis. Plant Cell Rep..

[37] Machado RD (2015). Comprehensive selection of reference genes for quantitative gene expression analysis during seed development in Brassica napus. Plant Cell Rep..

[38] Wang Y, Dai M, Cai D, Shi Z (2019). Screening for quantitative real-time PCR reference genes with high stable expression using the mRNA-sequencing data for pear. Tree Genet. Genomes.

[39] Zhang JR (2022). Systematic screening and validation of reliable reference genes for qRT-PCR analysis in Okra (*Abelmoschus esculentus* L.). Sci. Rep..

[40] Song Y, Hanner RH, Meng B (2021). Genome-wide screening of novel RT-qPCR reference genes for study of GLRaV-3 infection in wine grapes and refinement of an RNA isolation protocol for grape berries. Plant Methods.

[41] Ding A (2020). Screening of optimal reference genes for qRT-PCR and preliminary exploration of cold resistance mechanisms in Prunus mume and Prunus sibirica varieties. Mol. Biol. Rep..

[42] Du W, Hu F, Yuan S, Liu C (2019). Selection of reference genes for quantitative real-time PCR analysis of photosynthesis-related genes expression in Lilium regale. Physiol. Mol. Biol. Plants.

[43] Qi XY, Chen SS, Feng J, Deng YM (2020). Selection and validation of candidate reference genes for quantitative real-time PCR in *Jasminum sambac* Aiton. Acta. Agr. Boreali-Sinica..

[44] Lin, H., Qiu, B.Y., Zhang, Q.R., Li, D.Z. & Wen, Q.F. Screening and Evaluation of Reference Genes for qRT-PCR in Cauliflower (*Brassica oleracea* L. var. botrytis L.). *Mol. Plant Breed*. https://kns.cnki.net/kcms/detail/46.1068.S.20210914.1021.002. html (2021).

[45] Cui, Y.Q., Zhu, Z.B., Guo, Q.S., Lai, Q.J. & Xu, B.X. Screening of Internal Reference Genes by Quantitative Real-time PCR in *Sedum sarmentosum*. *Mol. Plant Breed*. https://kns.cnki.net/kcms/detail/46.1068.S.20220317.1731.024.html(2022).

[46] Yu XS (2021). Screening and stability evaluation of reference genes in *Uncaria rhynchophylla* qRT-PCR analysis. J. Agr. Biotechnol..

[47] Wang Y (2017). GSA: Genome Sequence Archive. Genom. Proteom. Bioinf..

[48] Li LS, Chen LN, Xia TZ, Yang HQ (2021). Selection of reference genes for quantitative real-time PCR analysis in *Cephalostachyum pingbianense*. Plant Physiol. J..

[49] Wu JY, He B, Du YJ, Li WC, Wei YZ (2017). Analysis method of systematically evaluating stability of reference genes using geNorm, NormFinder and BestKeeper. Mod. Agr. Sci. Tech..

[50] Kenneth JL, Thomas DS (2001). Analysis of relative gene expression data using real-time quantitative PCR and the 2^−ΔΔCT^ method. Methods.

