# Supplementary material for: Screening and validation of reference genes in Dracaena cochinchinensis using quantitative real-time PCR
Source: Sci Rep. 2024 Mar 14;14:6165. doi: 10.1038/s41598-024-52754-5 (PMC10940652; doi:10.1038/s41598-024-52754-5)
Supplement: Supplementary file 1 — Supplementary Information. [file 41598_2024_52754_MOESM1_ESM.pdf]

Supplementary Information

Screening and validation of reference genes in *Dracaena cochinchinensis* using Quantitative Real-Time PCR

Shixi Gao[a], Junxiang Peng[a], Mei Rong[a], Yang Liu[a], Yanhong Xu[a*], Jianhe Wei[a,b*]

1. **Statement:** *Dracaena cochinchinensis* was analyzed in this study and the formal identification of the plant materials was undertaken by Mr.J.H. Wei. We got the permission to collect the plant samples and all methods were performed in accordance with the relevant guidelines and regulations. The materials for qPCR are tissues from ten-year-old adult *D. cochinchinensis* trees growing in the germplasm bank at the Yunnan Branch of the Institute of Medicinal Plant Development, Chinese Academy of Medical Sciences, Jing-hong City (22.0058, 100.7885), China.

(This supplementary statement has been added to the manuscript.)

2.

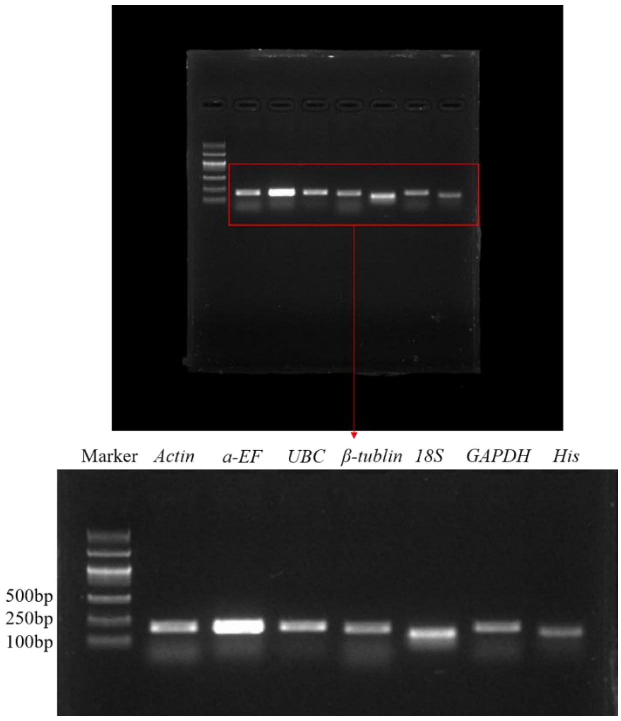

Supplementary Figure S1 Full-length gels of Fig.1A in manuscript

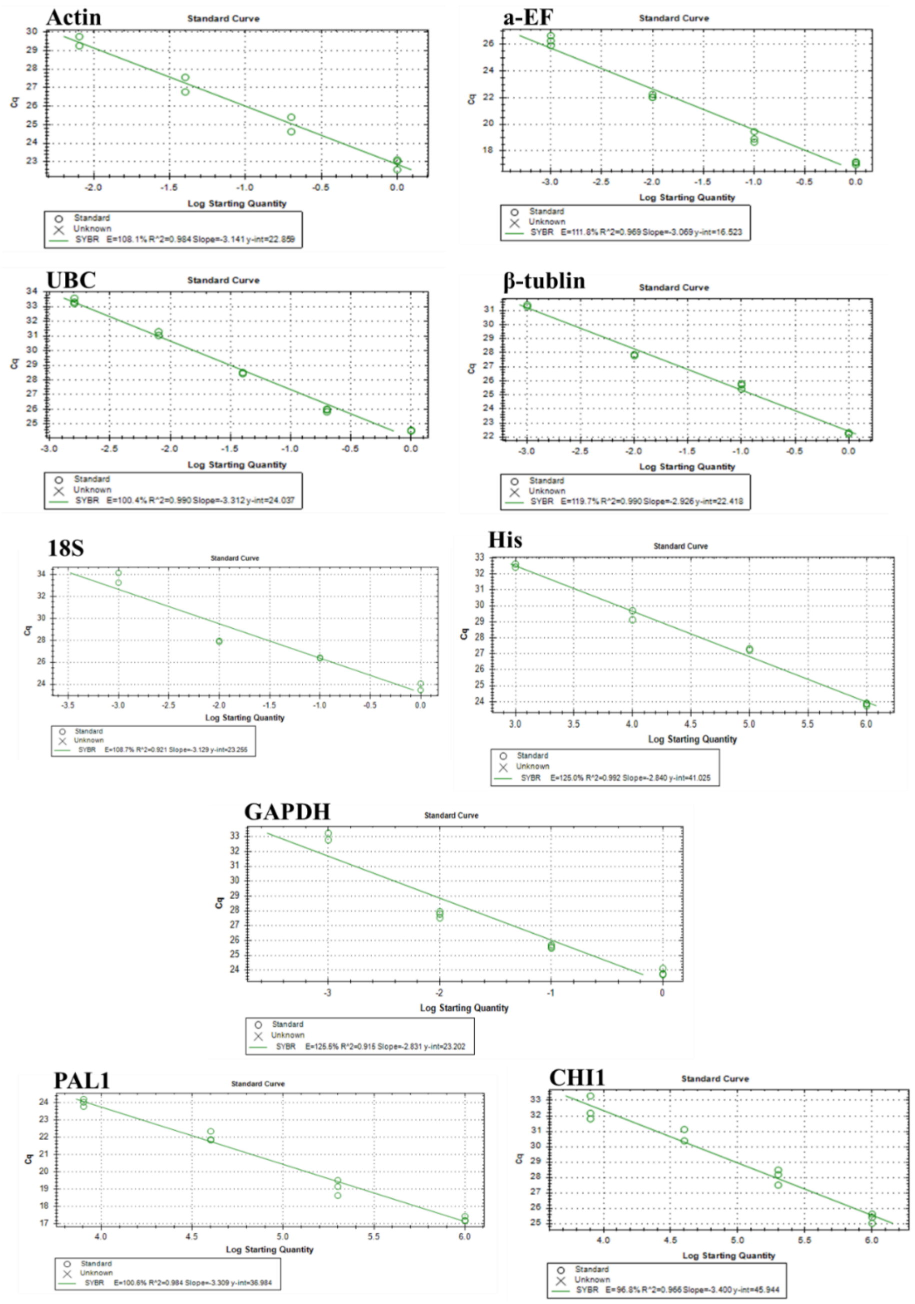

Supplementary Figure S2. Standard curve for amplification efficiency of candidate reference genes and target genes.

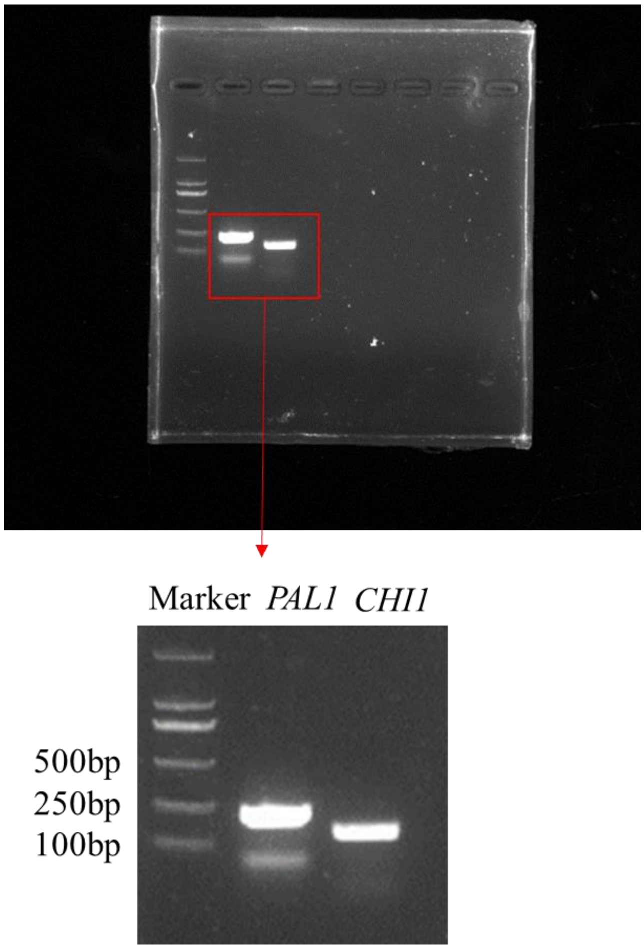

Supplementary Figure S3. Agarose gel electrophoresis of *PAL1* and *CHI1*.

Genes	Cq(NTC-1)	Cq(NTC-2)	Cq(NTC-3)
Actin	N/A	N/A	N/A
α-EF	43.96	36.62	39.13
UBC	N/A	N/A	N/A
β-tubulin	N/A	38.65	N/A
18S	33.46	34.79	33.50
GAPDH	N/A	N/A	N/A
His	N/A	N/A	N/A
PAL1	34.58	44.42	34.77
CHI1	43.75	45.57	43.48

Supplementary Table S1. Results for NTCs.
